# Integrating stomatal physiology and morphology: evolution of stomatal control and development of future crops

**DOI:** 10.1007/s00442-021-04857-3

**Published:** 2021-01-30

**Authors:** Matthew Haworth, Giovanni Marino, Francesco Loreto, Mauro Centritto

**Affiliations:** 1 National Research Council of Italy, Institute of Sustainable Plant Protection (CNR-IPSP), Via Madonna del Piano 10, 50019 Sesto Fiorentino, FI Italy; 2 Department of Biology, Agriculture and Food Sciences (CNR-DiSBA), National Research Council of Italy, Rome, Italy; 3 Department of Biology, University of Naples Federico II, Naples, Italy; 4 ENI-CNR Water Research Center “Hypatia of Alexandria”, Research Center Metapontum Agrobios, Metaponto, Italy

**Keywords:** Stomatal conductance, Stomatal density, Stomatal size, Stomatal kinetics, Stomatal anatomy

## Abstract

Stomata are central players in the hydrological and carbon cycles, regulating the uptake of carbon dioxide (CO_2_) for photosynthesis and transpirative loss of water (H_2_O) between plants and the atmosphere. The necessity to balance water-loss and CO_2_-uptake has played a key role in the evolution of plants, and is increasingly important in a hotter and drier world. The conductance of CO_2_ and water vapour across the leaf surface is determined by epidermal and stomatal morphology (the number, size, and spacing of stomatal pores) and stomatal physiology (the regulation of stomatal pore aperture in response to environmental conditions). The proportion of the epidermis allocated to stomata and the evolution of amphistomaty are linked to the physiological function of stomata. Moreover, the relationship between stomatal density and [CO_2_] is mediated by physiological stomatal behaviour; species with less responsive stomata to light and [CO_2_] are most likely to adjust stomatal initiation. These differences in the sensitivity of the stomatal density—[CO_2_] relationship between species influence the efficacy of the ‘stomatal method’ that is widely used to infer the palaeo-atmospheric [CO_2_] in which fossil leaves developed. Many studies have investigated stomatal physiology or morphology in isolation, which may result in the loss of the ‘overall picture’ as these traits operate in a coordinated manner to produce distinct mechanisms for stomatal control. Consideration of the interaction between stomatal morphology and physiology is critical to our understanding of plant evolutionary history, plant responses to on-going climate change and the production of more efficient and climate-resilient food and bio-fuel crops.

## An introduction to the origination and evolution of stomata—the importance of linking morphology and physiology

Stomata are tiny pores, ranging from 10 to 80 µm in length, that regulate leaf gas exchange by facilitating the diffusion of carbon dioxide (CO_2_) from the atmosphere to the chloroplast for photosynthesis (*P*_N_) and preventing excessive water-loss through transpiration. A stomatal complex is a pore enclosed by two guard cells, and in many plants surrounded by subsidiary cells (Edwards et al. 1998). Stomatal control is achieved via physiological regulation of guard cell turgor modifying stomatal pore aperture (Franks and Farquhar 2007), and morphological adjustment of the number and size of stomata on newly developing leaves (Woodward 1987). Stomata play a role in maintaining plant homeostasis, and represent an essential adaptive trait that has shaped plant evolutionary history (Robinson 1994; Haworth et al. 2011b; McAdam and Brodribb 2012b), and are a critical attribute in the development of more productive and ‘climate-proof’ food and biomass crops (Roche 2015; Lawson and Vialet-Chabrand 2019). In this review, we surmise that coordinated stomatal physiological and morphological responses operate in tandem to exert stomatal control. The majority of research has focused on stomatal physiology or morphology in isolation without consideration for co-occurring responses in the accompanying trait that can influence stomatal control. We show how the requirement to balance CO_2_-uptake against transpirative water-loss has generated a range of stomatal physiological and morphological strategies to regulate leaf gas exchange.

**Fig. 1:**
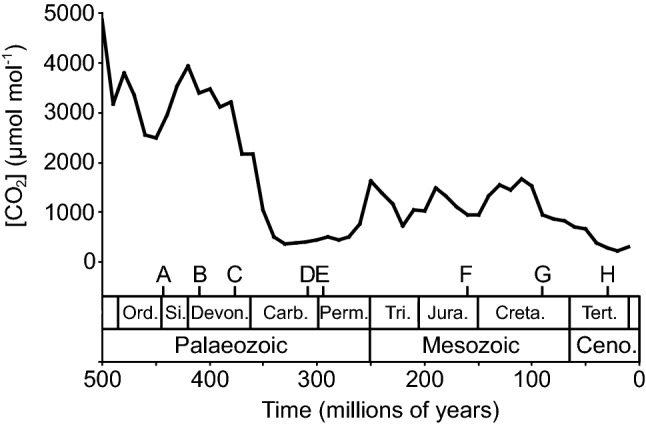
Biogeochemical modelled atmospheric [CO_2_] over the past 500 million years (Berner 2006, 2009). Letters indicate major events in plant evolutionary history. **a** Origination of vascular plants (Harrison and Morris 2018); **b** origination of stomata (Edwards et al. 1998; Duckett et al. 2009); **c** development of the planate leaf (Beerling et al. 2001); **d** origination of conifers (Leslie et al. 2018); **e** origination of cycads and ginkgoales (Tralau 1968; Pant 1987; Shen et al. 2005); **f** origination of angiosperms (Dilcher 2000; Soltis et al. 2008); **g** origination of C3 grasses/occurrence of phytoliths (Prasad et al. 2005; Strömberg 2011), and **h** origination of C4 grasses (Sage et al. 2012)

**Fig. 2:**
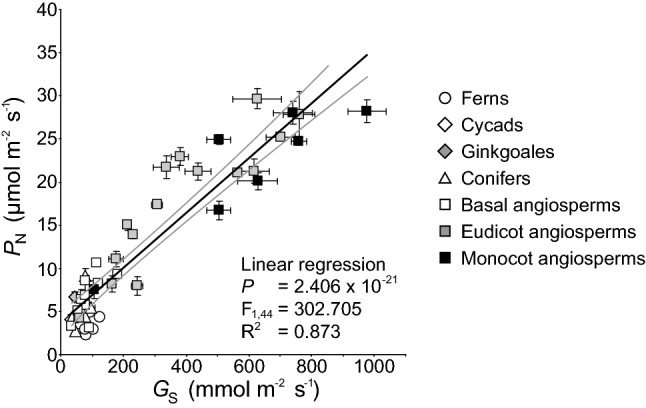
The relationship between photosynthesis (*P*_N_) and stomatal conductance (*G*_s_) in ferns, cycads, ginkgoales (*Ginkgo biloba*), conifers, basal angiosperms, eudicots and monocots (Haworth et al. 2018b). Data points are the mean and standard error of ≥ 5 replicates. The black line indicates the line of best fit and the two grey lines either side indicate the 95% confidence intervals of the mean

The earliest ‘stomata-like’ structures were not involved in gas exchange, but in the distribution of spores by allowing sporophyte tissues to dry more rapidly (Duckett et al. 2009). The evolutionary exaptation of these early stomata acted as a selective advantage by facilitating the diffusion of CO_2_ from the external atmosphere to the chloroplast (Chater et al. 2016). The earliest true stomata that originated ~ 410 million years ago (Fig. 1) are identical to their modern ‘kidney-shaped’ equivalents, indicating that their form and function has remained largely unaltered (Edwards et al. 1998). Stomatal conductance (*G*_s_) of CO_2_ (*G*_s CO2_) into the leaf and water vapour out of the leaf (*G*_s H2O_) occur simultaneously. As CO_2_ in the form of bicarbonate (HCO_3_−) is assimilated during photosynthesis, this creates a concentration gradient between the external atmosphere and the chloroplast following Fick’s law. The movement of CO_2_ experiences two main impeding resistance steps at the stomata and mesophyll layer. At the interface between the internal air-space and mesophyll, CO_2_ is hydrated to HCO_3_−, the moist surfaces of the mesophyll cells result in in the air within the leaf becoming more humid than the external atmosphere, inducing the diffusion of water vapour from the leaf through the stomatal pores (Cowan 1978; Harley et al. 1992). The costs and benefits associated with the requirement to exert stomatal control to balance CO_2_-uptake against water-loss has acted as an evolutionary driving force over Earth history (Fig. 1) (Robinson 1994; McAdam and Brodribb 2012b; Elliott-Kingston et al. 2016; Haworth et al. 2017b). The origination of many major groups of plants (Haworth et al. 2011b) and morphological and physiological developments such as the planate leaf (Beerling et al. 2001) and C4 photosynthesis (Monson 2003; Osborne and Beerling 2006; Sage et al. 2012) have coincided with declining or low atmospheric concentrations of carbon dioxide ([CO_2_]) (Fig. 1). Concomitant changes in factors such as temperature and/or water availability have induced selective pressures affecting photosynthesis and water use efficiency, specifically at the stomatal level, alongside [CO_2_] (Ehleringer and Monson 1993). Molecular evidence suggests the divergence of the angiosperms occurred during the Jurassic (201–145 Ma), but the expansion and diversification of the angiosperms occurred later during the Cretaceous (145–65 Ma) (Bell et al. 2005; Barba-Montoya et al. 2018) as [CO_2_] declined and [O_2_] rose decreasing rates of *P*_N_ relative to photorespiration (Fig. 1) (Haworth et al. 2017b). In extant plants, photosynthesis is positively related to *G*_s_ (Fig. 2), with the highest rates occurring in the more recently derived angiosperms, indicative of selective pressures favouring high rates of gas exchange. Those species with higher rates of *G*_s_ will require a greater proportion of the leaf epidermis to be devoted to stomata (*A*_%_) through higher stomatal density (SD) and/or stomatal size (SS). The selective pressures that lead to high *G*_s_ also render plants vulnerable to desiccation during episodes of low water availability or high evapotranspirative demand (Robinson 1994). Therefore, these selective pressures may also favour effective and rapid stomatal control (closure) to ensure plant survival during unfavourable conditions (Haworth et al. 2018b). Traits that confer a strong selective advantage rapidly become universal within a population (e.g. McNeilly 1968). However, a common stomatal control mechanism in terms of stomatal density, size, spacing and physiological behaviour is not apparent. The diversity of observed stomatal control mechanisms likely reflects trade-offs imposed by the interaction of factors such as habitat, water transport, leaf lifespan/economics and the legacy of evolutionary history. Moreover, it is worth bearing in mind that basal groups with stomatal physiological and morphological traits that are considered to be ‘more primitive’ are still successful today, indicating that selective processes do not act exclusively at the level of stomata and gas exchange but that other cost/benefits may determine the success of a species. In this paper, we will outline the evolution of physiological stomatal behaviour and stomatal morphology in terms of optimal allocation of the epidermis to gas exchange and optimality in stomatal behaviour. We will discuss their interaction in determining stomatal control, the implications for the use of the ‘stomatal method’ to reconstruct palaeo-[CO_2_], and the development of more productive and stress resistant crop varieties.

## Physiological stomatal behaviour

Opening and closing of the stomatal pore allows plants to regulate CO_2_-uptake and water-loss in response to the prevailing environmental conditions and the physiological status of the plant. Factors such as light, [CO_2_], leaf to air VPD, and plant water status interact to determine the degree of stomatal opening (Merilo et al. 2014; Ou et al. 2014; Haworth et al. 2018a). There are two main categories of physiological stomatal behaviour: active, where ions are pumped across the cell membrane to alter guard cell water potential and turgor, and passive, where guard cell water potential follows changes in whole leaf water status (Cowan 1978; Franks and Farquhar 2007; Ruszala et al. 2011). Regulation of the stomatal pore aperture is more rapid in species with active than passive stomatal behaviour (Brodribb and McAdam 2011; Doi et al. 2015; Haworth et al. 2015; Elliott-Kingston et al. 2016). The capacity to adjust *G*_s_ in response to fluctuating conditions, and thus optimise WUE over the short-term, may be considered a selective advantage (Cowan 1978). However, the lack of universality of active stomatal physiological behaviour may indicate costs associated with the capacity to detect and signal a shift in environmental conditions that is then manifested in the modulation of guard cell turgor. Selective pressures may not strongly favour optimal physiological stomatal behaviour in particular habitats and leaf lifespans as evidenced by the widespread persistence of plant groups and traits considered to be more basal. It is likely that the contrast between typically active and passive stomatal behaviour may be gradual rather than distinct (Haworth et al. 2013).

Photosynthetic processes are fundamentally driven by light (in the production of adenosine triphosphate, ATP, and reduced nicotinamide adenine dinucleotide phosphate, NADPH). Light is the central signal affecting stomatal opening / closing, with stomatal responses to factors such as high [CO_2_] and leaf to air VPD only occurring in the presence of light (Heath 1950; Shimazaki et al. 2007). The mechanisms underpinning stomatal opening in the light vary between species (Williams et al. 1983; Doi et al. 2015). Blue light stimulates stomatal opening by inducing the transport of potassium ions across the guard cell plasma membrane (Assmann and Shimazaki 1999). This pumping of potassium ions into the guard cells is observed in lycophytes, ferns, gymnosperms and angiosperms, suggesting that it originated in early plant lineages (Doi et al. 2015). However, the concentration of potassium ions in bryophytes during stomatal opening is not consistent with a flux from the subsidiary cells into the guard cells (Pressel et al. 2018). Stomatal opening is then sustained by red light driving *P*_N_ in the mesophyll which lowers [CO_2_] in the sub-stomatal internal air-space (*C*_i_) (Sharkey and Raschke 1981; Roelfsema et al. 2002); this maintains a constant ratio between *C*_i_ and the external atmospheric [CO_2_] (*C*_a_) under steady state conditions (Mott 1988). Photosynthesis within the guard cells may induce an increase in the concentration of malate (Ogawa et al. 1978; Shimazaki et al. 2007), and availability of ATP to pump ions into the guard cells (Tominaga et al. 2001; Suetsugu et al. 2014), resulting in stomatal opening as guard cell turgor increases. Guard cell chloroplasts are observed in most plant species (Zeiger et al. 2002) and are highly abundant in groups such as ferns (Doi and Shimazaki 2008), suggesting that they may play a fundamental role in maintaining stomatal opening in more basal groups. Darkness, or a reduction in the availability of light, induces a lowering of the concentration of osmolytes within the guard cell, reducing the water potential gradient between the guard cell and the surrounding subsidiary cells. The subsequent loss of turgor in the guard cells then causes a reduction in stomatal pore aperture (Shimazaki et al. 2007).

**Fig. 3:**
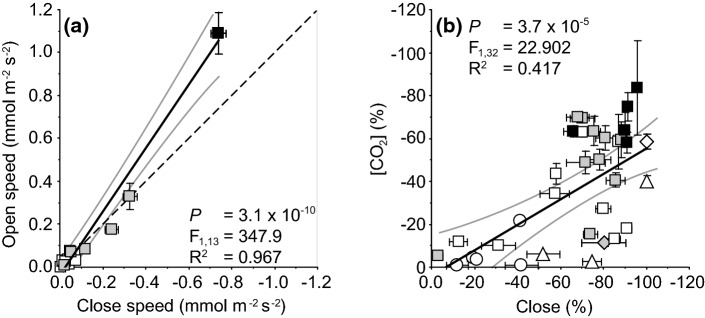
**a** The relationship between the rate of *G*_s_ increase (i.e. speed of stomatal opening) during a dark to light transition and the rate of *G*_s_ decrease (i.e. speed of stomatal closure) during a light to dark transition (see Fig. 4a and c for an example of stomatal kinetic responses). **b** Relationship between the percentage reduction in *G*_s_ during a step change in [CO_2_] from 400 to 2000 μmol mol^−1^ and the percentage reduction in *G*_s_ as stomatal close during a light to dark transition. Statistical analysis using linear regression. Data presented as in Fig. 1

A limitation of much analysis of stomatal physiological behaviour has been a lack of consistency between studies that has impeded comparability. It is noteworthy, that the speed of stomatal opening and closing are closely correlated (Fig. 3a) and broad patterns may be present in stomatal sensitivity to both light and [CO_2_] (Fig. 3b). It may then be possible to draw wider inferences from evolutionary studies of the underlying stomatal light response from the kinetics involved in both stomatal closure and opening. The speed of stomatal opening/closing (measured as the rate of change in *G*_s_ over the initial 50% of the *G*_s_ response: *G*_s50%_) and the extent of stomatal opening/closing (where closing can be expressed as the percentage closure or ‘tightness’: Fig. 3b) during a transition from dark to light (McAdam and Brodribb 2012b; Doi et al. 2015; Kardiman and Ræbild 2017; Xiong et al. 2018; Lawson and Vialet-Chabrand 2019; Lima et al. 2019), or light to dark (McAdam and Brodribb 2012b; Doi et al. 2015; Haworth et al. 2015, 2018b; Elliott-Kingston et al. 2016; Xiong et al. 2018) have been used to differentiate stomatal physiological response to light between plant groups with diverse evolutionary histories. It has been suggested that the evolutionary history of a species strongly affects its physiological stomatal behaviour (Elliott-Kingston et al. 2016; Hõrak et al. 2017). Plant groups that originated during episodes of low palaeo-atmospheric [CO_2_] (palaeo-[CO_2_]) exhibit faster rates of stomatal closure during a transition from light to dark conditions than groups that diverged when palaeo-[CO_2_] was higher (Fig. 1)(Elliott-Kingston et al. 2016). The speed of stomatal closure in basal angiosperms (such as *Amborella trichopoda*) is identical to rates observed in ferns and gymnosperms. The fastest rates of stomatal closure are found in more derived angiosperms, in particular the monocots (Haworth et al. 2018b). This increased stomatal responsiveness to light in the angiosperms may be correlated with differences in ratios of sucrose and malate acting as mesophyll derived signals regulating stomatal behaviour (Lima et al. 2019).

An instantaneous increase in atmospheric [CO_2_] favours *P*_N_ over photorespiration (Sharkey 1988; Tolbert et al. 1995; Haworth et al. 2017b). The stimulation in *P*_N_ associated with elevated [CO_2_] allows plants to lower *G*_s_ to maintain a constant *C*_i_: *C*_a_ ratio and increase WUE (Mott 1988; Eamus 1991; Franks and Beerling 2009a). This reduction in *G*_s_ can be achieved through decreases in stomatal pore aperture (Assmann 1999; Ainsworth and Rogers 2007), SD (Woodward 1987; Hu et al. 2019) or SS (Lammertsma et al. 2011; Haworth et al. 2016). Sub-ambient [CO_2_] (i.e. < 400 μmol mol^−1^) generally induces stomatal opening as higher *G*_s_ promotes diffusion of CO_2_ from the external atmosphere into the leaf (Heath 1948; 1950; Centritto et al. 2003). Experiments involving the separation of the epidermis and mesophyll layer suggest that guard cells sense and respond to sub-ambient [CO_2_] independently. However, stomatal closure as [CO_2_] rises above ambient requires physical contact between the epidermis and the mesophyll (the site of most *P*_N_ within the leaf) (Mott et al. 2008; Fujita et al. 2013); this is strongly indicative of a mesophyll derived signal such as bicarbonate sensed by carbonic anhydrases (Hu et al. 2010; Engineer et al. 2014). Instantaneous exposure to a range of [CO_2_] levels suggested that lycophytes, ferns, gymnosperms and angiosperms all showed stomatal opening at sub-ambient [CO_2_], but only angiosperms exhibited reduced *G*_s_ at [CO_2_] above ambient (Brodribb et al. 2009). This led to a hypothesis proposing that a transition from passive to active stomatal control in [CO_2_] and abscisic acid (ABA) sensitivity had occurred between the angiosperms and plant groups with more ancient evolutionary origins (Brodribb and McAdam 2011). The apparent divergence in stomatal CO_2_ sensitivity between the angiosperms and the lycophytes, ferns and gymnosperms may be linked to differences in calcium (Brodribb and McAdam 2013; Funk and Amatangelo 2013) and malate—sucrose (Lima et al. 2019) signalling. However, this evolutionary transition hypothesis was not supported by further gas exchange measurements that showed stomatal response to above ambient [CO_2_] in lycophytes, ferns and gymnosperms (Chater et al. 2011; Ruszala et al. 2011; Haworth et al. 2013, 2015; Franks and Britton‐Harper 2016; Hasper et al. 2017; Hõrak et al. 2017) and genetic analyses indicating that the genes responsible for [CO_2_] and ABA sensitivity occur in ancient plant lineages such as mosses and lycophytes (Chater et al. 2011, 2013; Ruszala et al. 2011; Lind et al. 2015; Cai et al. 2017).

Plant water status influences stomatal opening through hydraulic (leaf water potential) and chemical (such as plant hormones or changes in the pH of the xylem stream) signals (Wilkinson et al. 1998; Rodrigues et al. 2008; Tombesi et al. 2015; Brunetti et al. 2019). The timing and interaction of these chemical and hydraulic signals varies between species, affecting their response to reduced water availability. In the monocot grasses *Zea mays* (Tardieu et al. 1992) and *Arundo donax* (Haworth et al. 2017a) an increase in free-[ABA] is observed prior to any reduction in leaf water potential, characteristic of isohydric stomatal behaviours (Sade et al. 2012). In contrast, hydraulic signals precede chemical signals in *Populus nigra* (Marino et al. 2017), *Metasequoia glyptostroboides* (McAdam and Brodribb 2014), *Olea europaea* (Dbara et al. 2016) and *Vitis vinifera* (Correia et al. 1995; Tombesi et al. 2015). An increase in free-[ABA] surrounding the guard cell apoplast induces stomatal closure (Hartung 1983) by opening the SLAC1 anion channel and releasing ions such as potassium and chloride from the guard cell protoplast (Geiger et al. 2009). The increase in apoplastic free-[ABA] may be due to increased root to shoot transport via the xylem (Davies and Zhang 1991), enhanced conversion of inactive glucose-conjugated ABA stored in the vacuole to active free-ABA in the cytosol of cells within the leaf (Dietz et al. 2000; Seiler et al. 2011), reduced catabolism (Saito et al. 2004) and a promotion of synthesis in the leaves and stems of plants (Bauerle et al. 2004; Manzi et al. 2015; Brunetti et al. 2019). The interaction of hydraulic and hormonal signals determines stomatal response to soil drying. Stomatal [ABA] sensitivity is increased as leaf water potential falls (Tardieu and Davies 1992), and the retention of high concentrations of free-ABA within the leaf after re-watering maintains stomatal closure (Tombesi et al. 2015). Application of exogenous ABA to an evolutionary range of plants has produced contrasting results that have suggested that ABA sensitivity either developed in the angiosperms (Brodribb and McAdam 2011) or was acquired early in plant lineages (Ruszala et al. 2011; Grantz et al. 2019). Re-watering of plants after water deficit, when free-[ABA] levels in the leaf were still comparatively high (McAdam and Brodribb 2012a) and short-term foliar synthesis of free-ABA (McAdam and Brodribb 2015) supported interpretations of an evolutionary transition toward stomatal ABA sensitivity in the angiosperms. However, substitution of the open-stomata 1 (OST1) kinase which regulates the SLAC1 anion channel in ABA insensitive *Arabidosis thaliana* with the orthologue from the sporophyte of the moss *Physcomitrella patens* (which is not involved in gas exchange) restored ABA sensitivity in the *A. thaliana* mutants (Chater et al. 2011). Moreover, transcriptome analysis suggests that the proteins responsible for ABA signalling are present in mosses, lycophytes, ferns and angiosperms (Hanada et al. 2011; Lind et al. 2015; Cai et al. 2017). This discrepancy between observations of stomatal ABA sensitivity and insensitivity in ferns may be accounted for by their environment, with the relative humidity at which ferns are grown influencing the degree to which ABA affects *G*_s_ (Hõrak et al. 2017). It is noteworthy that while ferns exhibited ABA sensitivity, the extent and speed of the stomatal response was lower than that observed in the angiosperms (Hõrak et al. 2017; Grantz et al. 2019; Kübarsepp et al. 2020). Further analysis of the speed of stomatal responses under standardised conditions is required to explore any evolutionary patterns, characterise environmental influences on stomatal speed, classify the biochemical mechanisms regulating stomatal physiological behaviour in different plant groups, and identify the genes responsible for future crop development programs.

A wide variety of physiological stomatal behaviours are observed in response to light, [CO_2_], water availability, and leaf to air VPD. It is likely that evolutionary trends are present in the stomatal physiology of extant plants. However, it seems that this is unlikely to be on a binary ‘presence’ or ‘absence’ basis of stomatal physiological function (cf. Brodribb and McAdam 2011). The reality is almost certainly more complex and involves the interaction and influence of evolutionary trade-offs, acclimation to environmental/habitat conditions (including stress), ontogeny, and leaf economics. This picture may also be complicated by both ancient and modern plant groups that have ‘lost’ active stomatal physiological behaviours (Haworth et al. 2015; Hõrak et al. 2017) and questions of whether selective pressures have favoured optimality in certain habitats. Stomatal physiological behaviour is likely to be a combination of both ‘active’ and ‘passive’ responses (Franks 2013), as exemplified by the differing interactions of ABA and hydraulic signals in stomatal response to drought (Tardieu and Davies 1992; Tardieu et al. 1992; McAdam and Brodribb 2014; Tombesi et al. 2015; Brunetti et al. 2019).

## Stomatal morphology, density and size

**Fig. 4:**
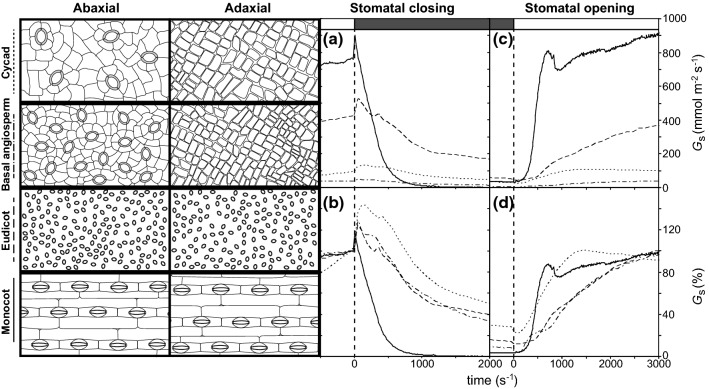
Example stomatal morphologies and distributions alongside stomatal closing (**a**, **b**) and opening (**c**, **d**) kinetics expressed as absolute and relative values for a cycad (*Cycas sinanensis*), a basal angiosperm (*Magnolia grandiflora*), a eudicot angiosperm (*Chenopodium quinoa*) and a monocot (*Arundo donax*). The key for identification of species is given on the left-hand *y*-axis. The rate of change of stomatal conductance, *G*_s_, during stomatal opening or closing (*G*_s50%_) is determined from the initial 50% of the *G*_s_ response

A key difference in stomatal morphology and physiological function is observed between the ‘dumb-bell’ stomata of the monocots and the ‘kidney-shaped’ stomata possessed by the majority of plants (Fig. 4) (Chen et al. 2017). The guard cells of dumb-bell stomata generally have a lower volume than kidney-shaped guard cells. This enables a greater relative turgor change when osmolytes are moved across the guard cell plasma membrane. Moreover, specialised subsidiary cells alongside the guard cells play a prominent role in moving osmolytes into the guard cells thus reducing their own turgor. This loss of turgor in the subsidiary cells allows the guard cells to expand, displacing the subsidiary cells. The mechanical advantage of the dumb-bell stomata along with their comparatively lower volume relative to surface area allow more rapid adjustments in stomatal pore area over larger pore areas (Franks and Farquhar 2007). This enables monocots to adjust their *G*_s_ more rapidly than species with kidney-shaped stomata (Fig. 4a–d) (Elliott-Kingston et al. 2016; McAusland et al. 2016; Haworth et al. 2018b). Despite the apparent advantages conferred by dumb-bell stomata (Haworth et al. 2018b), this adaptation is restricted to the Poaceae (Nunes et al. 2020). This may suggest that the origination of dumb-bell stomata is more complex than the evolution of other adaptations such as C4 metabolism that developed on numerous occasions (Sage et al. 2012), or that dumb-bell stomata incur selective costs. Nonetheless, this is a key area of interest in terms of improving the optimal performance of stomata in crop plants.

**Fig. 5:**
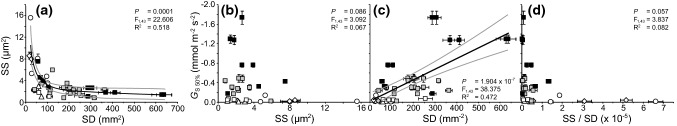
**a** The relationship between stomatal size (SS) and stomatal density (SD) (non-linear regression statistical analysis). The rate of *G*_s_ decrease during a light to dark transition (*G*_s50%_) versus **b** SS (linear regression), **c** SD (linear regression) and **d** the SS:SD ratio (linear regression). Data presented as in Fig. 2 (data from Haworth et al. 2015, 2018b)

The number, size, and distribution of stomata determine the limits for stomatal physiological adjustment (Drake et al. 2013; Haworth et al. 2013; Kardiman and Ræbild 2017), and are correlated to modelled (de Boer et al. 2011; Dow et al. 2014; McElwain et al. 2016) and measured rates of *G*_s_ (Haworth et al. 2018b; Xiong and Flexas 2020). An inverse relationship is found between SD and SS in both living and fossil plants (Hetherington and Woodward 2003; Franks and Beerling 2009b; Lammertsma et al. 2011; de Boer et al. 2016; Haworth et al. 2018b) (Fig. 5a). This SD/SS relationship may be a simple reflection of geometry, in that it is not possible to fit high densities of large stomata over a leaf surface due to space constraints and the effect on the structural integrity of the leaf (at least one epidermal cell generally separates stomatal complexes: Peterson et al. 2010), and are coordinated with the structure of the mesophyll (Lundgren et al. 2019; Baillie and Fleming 2020). Nevertheless, evidence suggests that the inverse relationship between SD and SS may also have an adaptive significance. More ancient plant lineages such as ferns, lycophytes and cycads generally exhibit lower densities of large stomata, while high densities of small stomata are generally found in the angiosperms (Fig. 5a). This has been attributed to the palaeo-[CO_2_] in which specific plant groups originated (Franks and Beerling 2009b). High densities of small stomata are considered to shorten the diffusion distance for CO_2_ from the external atmosphere to the chloroplast and maximise the potential for gas exchange for a given *A*_%_, thus serving as a selective advantage for angiosperms that originated in comparatively lower palaeo-[CO_2_] (Franks and Beerling 2009b; de Boer et al. 2016). Combined physiological and morphological analysis is required to assess whether a high SD/SS ratio does in fact serve as a selective advantage depending upon the growth strategy of the species (e.g. leaf lifespan and investment), environmental factors (e.g. [CO_2_], atmospheric pollution) and biotic factors (such as pathogen entry via the stomata: Muir 2015) given the persistence and continued evolutionary success of more ancient species with comparatively low SD/SS ratios. Study of within species changes in the SD/SS relationship and any potential functional significance of shifts in the ratio of SD to SS is also required. The arrangement of stomata over a leaf surface is also reliant upon the water transport capacity of the leaf. An increase in the complexity of leaf veins may have enabled eudicot angiosperms to support higher numbers of small stomata over the leaf surface (Brodribb and Feild 2010; de Boer et al. 2012) alongside the generally greater conductivity of xylem vessels compared to tracheids (Sperry et al. 2007).

Stomatal density, size, and distribution are set during leaf development (e.g. Lake et al. 2001; Šantrůček et al. 2014). The determination, division, and expansion of cells into epidermal pavement, subsidiary or guard cells is regulated by a series of genes (SPEECH, MUTE, and FAMA) (MacAlister et al. 2007; Zoulias et al. 2018). Mutants of the moss *P. patens* lacking the genes that encode these transcriptome regulators lacked stomata-like structures in the sporophylls, suggesting that the genetic apparatus to regulate stomatal patterning originated in early plant lineages (Chater et al. 2016). Manipulation of these same genes enabled the development of rice (*Oryza sativa*) varieties with 50–80% lower SD values than the unaltered control. Under elevated [CO_2_] the rice genotypes with lower SD exhibited enhanced tolerance to drought but this was not apparent at ambient [CO_2_] (Caine et al. 2019) indicating that optimality may be favoured by the pressures exerted under specific environmental conditions. However, while reducing SD in crops may decrease water-loss it will also limit CO_2_-uptake for *P*_N_ (Bertolino et al. 2019), the potential of a crop to exploit episodes favourable to *P*_N_ (McAusland et al. 2016; Haworth et al. 2018b), the capacity to generate root-mass flow for the uptake of mobile nutrients (Van Vuuren et al. 1997; Caird et al. 2007), and the potential for evapotranspirative cooling (Jones 1999; Beerling et al. 2001). These are critical constraints that should be considered in any attempt to adjust the stomatal morphology of crop plants without consideration of stomatal physiological behaviour.

The distribution of stomata over the leaf surface also plays an important role in the capacity for leaf gas exchange. The majority of plant species possess stomata on the abaxial leaf surface (hypostomatous distribution) (Salisbury 1927; Peat and Fitter 1994; Muir 2015). Hypostomaty is considered to represent the primitive state of stomatal distribution, as an increased incidence of amphistomaty in more derived plants is indicative of an evolutionary trend (Mott et al. 1982). Amphistomatous species possess stomata on the abaxial and adaxial leaf surfaces. Utilising both leaf surfaces increases the potential for leaf gas exchange and reduces the impact of mesophyll limitations on *P*_N_ (Parkhurst 1978; Mott et al. 1982; Peat and Fitter 1994; Muir 2018; Xiong and Flexas 2020). The majority of amphistomatous species exhibit high rates of *P*_N_ and occupy high light environments suited to rapid growth (Parkhurst 1978; Mott et al. 1982; Haworth et al. 2018b; Muir 2018). This suggests that higher *P*_N_ through enhanced capacity for gas exchange may have acted as a selective pressure favouring the development of amphistomaty (Mott et al. 1982) (Fig. 2). However, there may be some selective costs associated with amphistomaty. Increasing the allocation of the entire leaf surface for gas exchange may render plants more vulnerable to excessive water-loss during periods when water availability is low and/or evapotranspirative demand high. In the case of species such as *Olea europaea* occupying arid environments, where for extended periods water availability limits growth to a greater extent than the potential for photosynthetic CO_2_-uptake, minimising water-loss by largely restricting gas exchange to one surface may be advantageous (Guerfel et al. 2007). Amphistomatous species may also be more vulnerable to infection via pathogenic fungi which enter the leaf through stomata (Muir 2015) and higher stomatal conductance is associated with increased entry of toxic atmospheric gases (Hoshika et al. 2020). The majority of amphistomatous species have equal distributions of stomata on the abaxial and adaxial surfaces (so-called ‘perfect’ amphistomaty), suggesting that the selective pressures acting on stomatal distribution tend to strongly favour either ‘optimal outcomes’ of amphistomaty or hypostomaty with little evolutionary benefit for partial amphistomaty (Muir 2015).

## Optimal allocation of the epidermis and stomatal kinetics

**Fig. 6:**
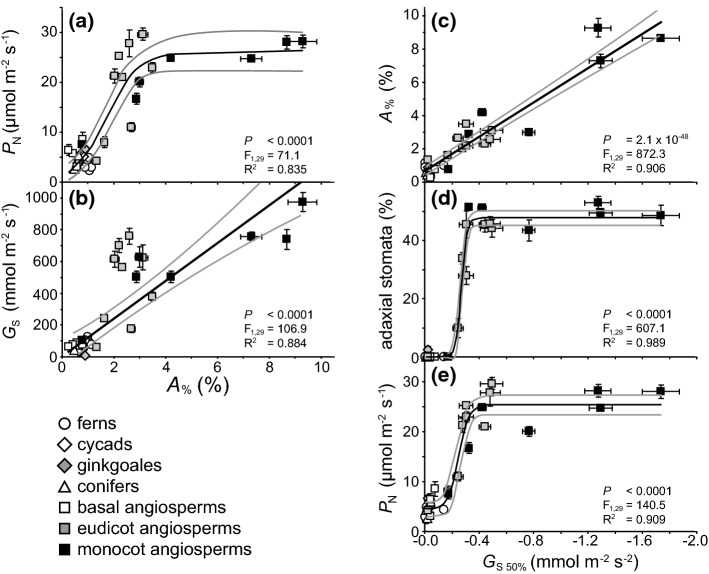
The relationships between *A*_%_ to *P*_N_ **a** (non-linear regression) and *G*_s_, **b** (non-linear regression). The rate of *G*_s_ decrease during a light to dark transition (*G*_s50%_) versus *A*_%_, **c** (linear regression), the percentage of stomata on the adaxial leaf surface, **d** (non-linear regression) and *P*_N_, **e** (non-linear regression). Presented as in Fig. 2 (data from Haworth et al. 2018b)

The greater the allocation of the epidermis as stomata, the higher the capacity for CO_2_− uptake but also the potential for water-loss. Modifying plants to possess lower SDs may be a successful approach to enhancing drought tolerance by decreasing *G*_s_ (Hepworth et al. 2015; Bertolino et al. 2019; Caine et al. 2019); however, restricting maximum *G*_s_ constrains the ability of plants to take-up CO_2_ and fully exploit transient conditions favourable to growth (Fig. 6a and b). As outlined earlier, increasing stomata density has evolutionary value in a ‘low [CO_2_] world’, but also exposes plants to negative consequences when encountering stress. Plants with both highly physiologically functional stomata (i.e. the capacity to adjust stomatal pore aperture rapidly) and a large proportion of the epidermis allotted to stomata may be most suited to improve crop productivity and WUE (e.g. Haworth et al. 2018a; Durand et al. 2019). Across a diverse evolutionary range of plants, the speed of stomatal closure (during a transition from saturating light conditions to darkness: e.g. Fig. 4a and b) was positively correlated with the proportion of *A*_%_ (Fig. 6c) (this relationship was apparent utilising both absolute and normalised values of *G*_s50%_). The highest rates of *G*_s50%_ adjustment and values of *A*_%_ were found in the monocot angiosperms, suggesting an evolutionary trajectory favouring more responsive stomata and higher *A*_%_, possibly in response to selective pressures induced by declining Cenozoic [CO_2_] (Fig. 1). Those species with more responsive stomata possess stomatal complexes evenly distributed across the entire epidermis (Fig. 6d). The capability to utilise both leaf surfaces for gas exchange (Fig. 6a) was accompanied by increased stomatal function with a comparatively tight transition observed between hypostomatous and amphistomatous distributions and the speed of stomatal closure (Haworth et al. 2018b). This is consistent with observations of a bimodal split in stomatal distribution between perfect hypostomaty or amphistomaty (Muir 2015). The evidence would suggest that allocating a high proportion of the epidermis to stomata is not viable unless accompanied by highly responsive physiological stomatal behaviour (Fig. 6c). It can be envisaged that selective pressures have acted to favour both increased physiological stomatal control (Franks and Farquhar 2007; Raven 2014) and greater *A*_%_ (Franks and Beerling 2009b; de Boer et al. 2016) in unison (Haworth et al. 2018b). The fast growing monocot *A. donax* possesses one of the highest *A*_%_ values and extremely responsive stomata (Fig. 4). Moreover, under conditions of drought stress, as the concentration of free-ABA within the leaf rises, the stomata of *A. donax* become increasingly sensitive to changes in light intensity and CO_2_ availability (but not leaf to air VPD) (Haworth et al. 2018a). Similar increases in stomatal sensitivity to light have been observed in *Nicotiana tabacum* (Gerardin et al. 2018), *Populus euramericana* and *Populus nigra* (Durand et al. 2019) grown under water deficit. Increasing the sensitivity of guard cells to free-ABA could further enhance the functionality of stomata to optimise *P*_N_ and WUE over the short-term (Mega et al. 2019). The stomatal patterning and function of a species such as *A. donax* may serve as a useful ideotype in maximising photosynthetic gain during optimal conditions, but also tolerance to drought through effective physiological stomatal control. Phenotyping of species with high *A*_%_ and *G*_s50%_ will enable characterisation of attributes conducive to high *P*_N_ and optimal stomatal behaviour alongside the identification of genes that underpin these traits to develop more productive and climate resilient crops.

Higher densities of small stomata are not only considered to represent a selective advantage by reducing the diffusion distance for CO_2_-uptake (de Boer et al. 2016), but also respond more rapidly to external stimuli. The lower surface area to volume ratio of smaller guard cells is proposed to allow more rapid fluxes of ions into and out of the guard cell plasma membrane, thus enabling faster and more sensitive adjustment of pore aperture and *G*_s_ (Raven 2014; Lawson and Vialet-Chabrand 2019). Higher densities of smaller stomata were associated with faster rates of *G*_s_ increase during stomatal opening of five *Banksia* (Drake et al. 2013), eleven rainforest eudicots (Kardiman and Ræbild 2017), two *Populus* (Durand et al. 2019) and 16 pteridophyte (Kübarsepp et al. 2020) species. However, across a more diverse range of plants (ferns, cycads, *Ginkgo biloba*, conifer and eudicot/monocot angiosperms) with differing stomatal morphologies and physiological behaviours the rate of *G*_s_ decrease during stomatal closure was not related to SS (Fig. 5b) (Elliott-Kingston et al. 2016; Haworth et al. 2018b) or the SS:SD ratio (Fig. 5d), but was positively correlated to SD (Fig. 5c) (Haworth et al. 2018b). Analysis of *G*_s_ to a step change from low to high and back to low light suggested that the speed of stomatal response was related to size in dumb-bell but not kidney-shaped guard cells (McAusland et al. 2016). In closely related plants with similar physiological stomatal control, morphology likely plays a significant role in determining stomatal function (e.g. Drake et al. 2013). It may be hypothesised that stomatal control is determined by the interaction of stomatal morphology (e.g. Woolfenden et al. 2018) and physiology (e.g. Brodribb and McAdam 2011), and the respective contributions of morphology or physiology to stomatal control likely varies between species (Haworth et al. 2018b).

Increased yield achieved through traditional breeding programs has been accompanied by higher *G*_s_ (Roche 2015), but not necessarily enhanced stomatal function and WUE (e.g. Lauteri et al. 2014). This raises the possibility that further selection on the basis of optimal stomatal physiological behaviour and enhanced allocation of the epidermis may be effective in promoting yield and stress tolerance. The optimisation of stomatal control is extremely complex, resulting in a range of strategies that vary depending upon the growth conditions (Gerardin et al. 2018; Haworth et al. 2018a; Durand et al. 2019). Nonetheless, greater *A*_%_ requires more responsive stomata (Fig. 6c) (Haworth et al. 2018b). This relationship has likely played a central role in plant evolutionary history (Haworth et al. 2011b), and also informs the traits required to underpin productive and climate proof crops in the future (Haworth et al. 2018a; Faralli et al. 2019). Identification of the quantitative trait loci that encode stomatal pattering and physiological function should be a priority in developing crops with the epidermal patterning and stomatal morphological/physiological traits required to complement enhanced biochemical photosynthetic efficiency (e.g. Leegood 2013).

## Stomatal responses to [CO_2_] and implications for the stomatal method of Palaeo-[CO_2_] reconstruction

**Fig. 7:**
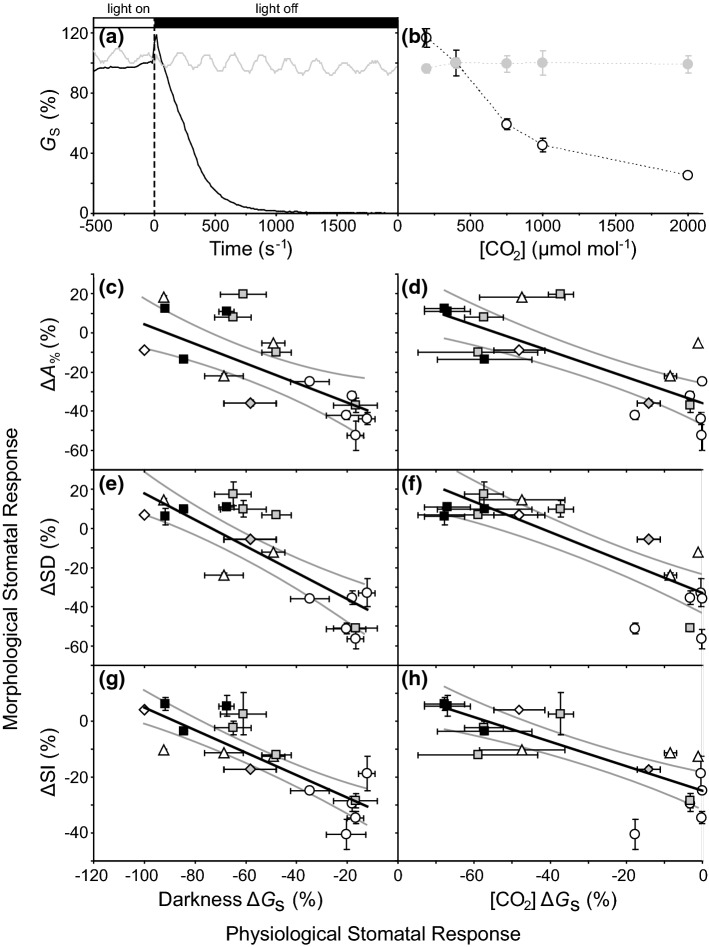
Typical responses of species with active (black line and black data points) and passive (grey line and grey data points) physiological stomatal behaviour to **a** a transition from light to dark and **b** a step increases in [CO_2_] from 200 to 2000 μmol mol^−1^ (*G*_s_ is expressed as a percentage relative to the point where illumination was ended at time 0 and *G*_s_ at 400 μmol mol^−1^). The relative change in epidermal and stomatal morphological parameters in plants grown in ambient (400 μmol mol^−1^) and extremely elevated (1500 or 2000 μmol mol^−1^) [CO_2_] versus the physiological *G*_s_ response to darkness (darkness ∆*G*_s_) or a change in [CO_2_] from 400 to 2000 μmol mol^−1^ ([CO_2_] ∆*G*_s_): **c**) ∆*A*_%_ versus darkness ∆*G*_s_ (linear regression *P* = 0.0007.517 × 10^–4^; *F*_1,19_ = 16.067; *R*^2^ = 0.458); **d** ∆*A*_%_ versus [CO_2_] ∆*G*_s_ (linear regression *P* = 5.718 × 10^–5^; *F*_1,19_ = 26.502; *R*^2^ = 0.582); **e** ∆SD versus darkness ∆*G*_s_ (linear regression *P* = 2.489 × 10^–6^; *F*_1,19_ = 43.771; *R*^2^ = 0.697); **f** ∆SD versus [CO_2_] ∆*G*_s_ (linear regression *P* = 7.053 × 10^–6^; *F*_1,19_ = 37.383; *R*^2^ = 0.663); **g** ∆SI versus darkness ∆*G*_s_ (linear regression *P* = 7.754 × 10^–7^; *F*_1,19_ = 51.812; *R*^2^ = 0.7316), and; **h** ∆SI versus [CO_2_] ∆*G*_s_ (linear regression *P* = 2.455 × 10^–5^; *F*_1,19_ = 30.608; *R*^2^ = 0.617). Presented as in Fig. 2 (data from Haworth et al. 2013, 2015; Elliott-Kingston et al. 2016).

As the substrate for *P*_N_, the availability of CO_2_ in the atmosphere exerts a strong influence on leaf gas exchange (Fig. 1). As [CO_2_] increases, *G*_s_ generally declines. Reduced *G*_s_ can be directly achieved in the short-term by physiological stomatal closure (Jarvis et al. 1999; Centritto et al. 2003) and an acclimation response (Centritto et al. 1999) over the longer term by reductions in SD (Woodward 1987) that possibly develops into an adaptation over multiple generations (Bettarini et al. 1998; Watson-Lazowski et al. 2016). Indeed, the inverse correlation between SD or stomatal index (SI: a normalised ratio of epidermal cells to stomata which gauges stomatal initiation) and [CO_2_] is one of the most well-established relationships in botany (Woodward 1987; Beerling and Chaloner 1993a; Woodward and Kelly 1995; Beerling and Kelly 1997), and has been utilised extensively to infer the palaeo-[CO_2_] in which fossil leaves developed (e.g. Passalia 2009; Smith et al. 2010; Jing and Bainian 2018; Steinthorsdottir et al. 2019). Species specific SD and SI responses (in both occurrence and extent) to the availability of CO_2_ can be assessed by analysis of the number of stomata and epidermal cells in the leaves of historical herbarium specimens collected during the last ~ 250 years as [CO_2_] has risen from 280 to above 400 μmol mol^−1^, [CO_2_] enrichment studies, and over altitudinal gradients where the partial pressure of CO_2_ (*p*CO_2_) varies (but the concentration of CO_2_ remains constant, uncoupling the effect of [CO_2_] from CO_2_-availability) (Woodward 1987; Woodward and Bazzaz 1988; Beerling and Chaloner 1993b; Kürschner et al. 1997, 2008; Kouwenberg et al. 2003; Haworth et al. 2010; Lammertsma et al. 2011; Hu et al. 2019). However, the SD and SI response to [CO_2_] varies between species in the occurrence of any relationship (some plant groups such as the cycads do not alter SD or SI to [CO_2_] and are known as ‘SD non-responders’: Haworth et al. 2011c), the extent of the SD or SI response and the [CO_2_] range over which SD or SI responds (Beerling and Chaloner 1993a; Kürschner et al. 1996; Kürschner 1997; Haworth et al. 2013; Hu et al. 2015; Hill et al. 2019). For example, many angiosperms alter SD and SI to [CO_2_] below 400 μmol mol^−1^, but reach a ‘ceiling of response’ at [CO_2_] levels above current ambient (Kürschner et al. 1996; Kürschner 1997). In contrast, many conifers continue to reduce SD and SI at [CO_2_] above 400 μmol mol^−1^ (Haworth et al. 2011a). This pattern may be associated with the generally more active physiological behaviours observed in the angiosperms resulting in less pronounced morphological responses to elevated [CO_2_] (Haworth et al. 2013, 2015). An inherent weakness in the ‘stomatal method’ of reconstructing palaeo-[CO_2_] is that it is not possible to determine whether an extinct fossil plant was a SD responder or not. One possible explanation for the variation in SD and SI responses to [CO_2_] is the interaction between stomatal morphology and physiology in determining stomatal control. As described earlier, physiological stomatal behaviours can be categorised as ‘active’ or ‘passive’. Figure 7a and b show typical active (the black line and data points) and passive (the grey line and data points) *G*_s_ responses to a light to dark transition and step increases in [CO_2_]. When grown in atmospheres of elevated [CO_2_], those plants with active physiological stomatal behaviour generally show lower SD, SI and *A*_%_ responses on newly developed leaves than their counterparts with passive physiological stomatal behaviour (Fig. 7c–h). It is possible to infer two stomatal control strategies to [CO_2_]; ‘passive stomatal behaviour/SD responders’ and ‘active stomatal behaviour/SD non-responders’ (Haworth et al. 2015). However, the SD and SI response to [CO_2_] is not clearly bimodal (as in the occurrence of perfect hypostomaty and amphistomaty), with species exhibiting a range of responses along these two extremes (e.g. Fig. 7g), consistent with observations that many plants utilise combinations of both active and passive physiological stomatal behaviour (Franks 2013; Brunetti et al. 2019). Stomatal physiology and morphology operate in tandem to determine stomatal control in response to [CO_2_] (Haworth et al. 2015). This relationship is key to predictions of stomatal and transpirative responses to [CO_2_] (e.g. Ball et al. 1987; Gao et al. 2002; Medlyn et al. 2011). Consideration should also be given to the efficacy of modelling maximum *G*_s_ based on stomatal morphological parameters alone (in particular when the presence of stomatal occlusions such as wax plugs are neglected: e.g. McElwain et al. 2016).

**Fig. 8:**
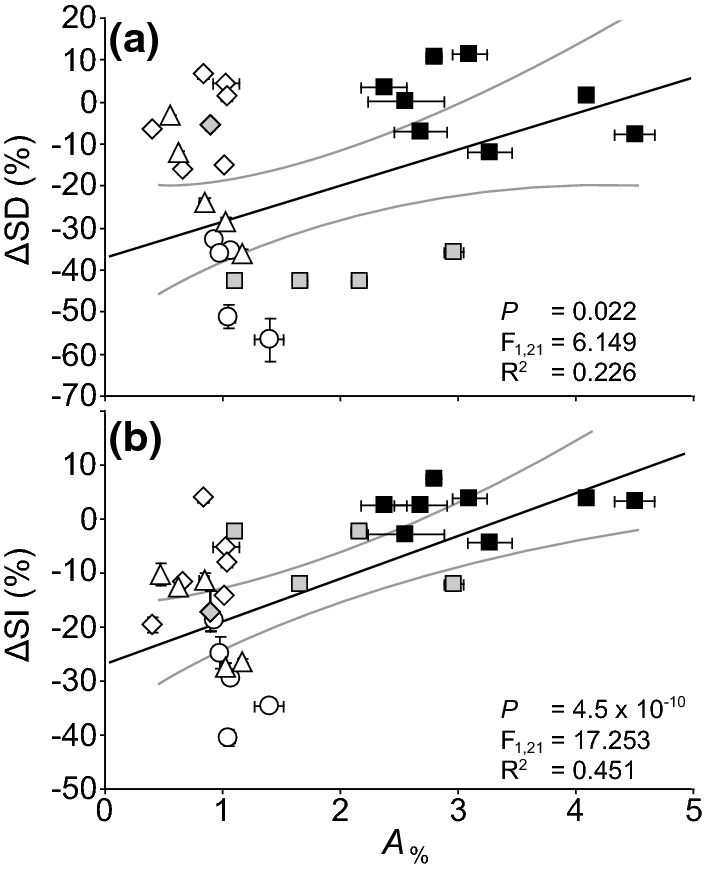
The relative change in **a** SD (∆SD) and **b** SI (∆SI) of an evolutionary range of plants in leaves developed in ambient (400 μmol mol^−1^) and extremely elevated (1500 or 2000 μmol mol^−1^) [CO_2_] in relation to the *A*_%_ values of plants grown at ambient [CO_2_]. The figure shows the response of cycads (diamond symbol white fill); however, cycads were not included in the regression analysis as cycads do not alter stomatal initiation in response to [CO_2_]. Data indicates the results of linear regression analysis. Presented as in Fig. 2 (data from Haworth et al. 2011a, c, 2013, 2015)

Active and passive physiological stomatal behaviours have been demonstrated in a wide range of plant groups (Brodribb et al. 2009; Brodribb and McAdam 2011; Ruszala et al. 2011; Haworth et al. 2015). The wax cuticle is frequently the only structure preserved in fossil plants (e.g. Oldham 1976; Watson 1977; Carrizo et al. 2019). From this ‘exoskeleton’ of the leaf it is not possible to demonstrate the type of physiological stomatal behaviour exhibited by a fossil plant (Haworth et al. 2013), as even closely related species exhibit contrasting physiological behaviours, and many plants have ‘lost’ the capacity for active stomatal physiology (Doi et al. 2015; Hõrak et al. 2017). However, in light of the observation that *A*_%_ is strongly related to stomatal responsiveness (Fig. 6c), it may be possible to infer the likelihood of a fossil plant being a SD responder on the basis of *A*_%_. If cycads are excluded (as a group cycads exhibit low *A*_%_ and do not alter SD: Haworth et al. 2011c), it is possible to observe that species with an *A*_%_ around 1.0–1.5% are more likely than species with an *A*_%_ above 2.0% to alter stomatal initiation in response to an increase in [CO_2_] (Fig. 8). The lack of a SD response in the cycads may be associated with their origination during a period of comparatively high-[CO_2_] combined with an already low SD that reduces the potential of further SD adjustment and enable a minimum rate of leaf gas exchange (Haworth et al. 2011c). The apparent relationship between *A*_%_ and the relative change in SI is more robust than that of SD. This may reflect the more variable nature of SD as leaf expansion may be affected under elevated [CO_2_]. It should be stressed that this relationship is built upon an assumption, and may not apply to distantly related extinct fossil plants. However, palaeobotanists may assess the *A*_%_ of fossil plants to gauge the likelihood of the SD and SI values of their target species reflecting the palaeo-[CO_2_] in which the leaf developed. Further [CO_2_] enrichment studies should assess possible relationships between *A*_%_ and the responsiveness of stomatal initiation to [CO_2_] more robustly, alongside material analysis of guard cell structures to determine their likely physiological function (e.g. Carter et al. 2017; Woolfenden et al. 2018) to refine the stomatal palaeo-[CO_2_] method by identifying traits likely to indicate whether or not a fossil plant was a SD-responder.

## Conclusions

Plant gas exchange is crucial to plant growth, survival during abiotic stress, and the cycling of CO_2_ and water. Stomatal conductance is determined by the interaction of stomatal physiological behaviour and stomatal morphology. However, stomatal physiology and morphology have too often been considered in isolation. Here, we have shown that the coordination of stomatal morphology and physiology has played a central role in plant evolution, allowing the angiosperms to exploit more of the leaf epidermis for photosynthetic CO_2_-uptake (Fig. 6) and shaped plant responses to atmospheric [CO_2_] (Fig. 7). Increased physiological stomatal functionality, such as the origination of dumb-bell stomata, will inevitably effect stomatal morphology. Indeed, the selective pressures acting upon stomatal physiological behaviour also influence stomatal morphological adaptation. The diverse range of stomatal control strategies observed likely reflect trade-offs between the selective costs and benefits involved in exerting stomatal control in a multitude of environments and the investment in each leaf. As [CO_2_] increases and temperatures rise globally alongside more frequent droughts in semi-arid and arid regions, an understanding of stomatal control will be fundamental to the development of more productive climate resilient crops. Modification of stomatal morphology (Bertolino et al. 2019; e.g. Harrison et al. 2020) or physiology (e.g. Mega et al. 2019) in isolation is unlikely to achieve the ideotype characteristics of an ability to exploit favourable growth conditions but also withstand abiotic stress. High *A*_%_ accompanied by highly functional and responsive stomata would reflect optimal stomatal control for any fast growing crop species cultivated in drought prone areas. Analysis of fast growing and drought resistant eudicots and monocots may enable the synthesis of stomatal physiological and morphological research towards developing enhanced stomatal control in future crops.
